# BRCA1 and BRCA2 mutations in Scotland and Northern Ireland

**DOI:** 10.1038/sj.bjc.6600840

**Published:** 2003-04-15

**Authors:** The Scottish/Northern Irish BRCA1/BRCA2 Consortium

**Affiliations:** 1 Bute Medicine, University of St Andrews, St Andrews, KY16 9TS, Scotland, UK

**Keywords:** breast cancer, familial, BRCA1, BRCA2, Scotland, Northern Ireland

## Abstract

BRCA1 and BRCA2 mutations have been identified in 107 families in Scotland and Northern Ireland. Ninety-seven of these families had been referred to regional cancer genetics centres and a further 10 were identified from a sequential series of male breast cancers treated in Edinburgh. Fifty-nine of the families had a mutation in BRCA1 and 46 in BRCA2. Two families had both. Family trees were extended and cancer diagnoses verified by means of the unusually complete and accessible Scottish and Northern Irish records. Ten specific recurring mutations (five in each gene) accounted for almost half of the total detected, and almost one-quarter were accounted for by just two (BRCA1 *2800 delAA* and BRCA2 *6503 delTT*). The prevalence of breast cancer is similar for BRCA1 and BRCA2 mutation families (average 3.7 and 3.6 per family), but the former have a much greater risk of ovarian cancer (average 1.5 and 0.6 per family, respectively). For breast cancer, age of onset tended to be younger in BRCA1 mutation carriers but, for ovarian cancer, there was no difference between BRCA1 and BRCA2 families in mean age at diagnosis. Mutations within the 5′ two-thirds of BRCA1 carry a significantly higher relative risk of ovarian cancer and the same is true for mutations within the central portion of BRCA2 (the ‘OCCR’). In the former case, this appears to be because of differences in absolute risk for both ovarian and breast cancer, while, in the latter, only ovarian cancer risk varies significantly. The findings confirm that founder mutations are present within the Scottish/Northern Irish population and have implications for the organisation of molecular screening services.

Worldwide variation in the distribution of BRCA1 and BRCA2 mutations is well recognised (Szabo and King, 1997). In several populations or ethnic groups, distinctive founder mutations form a sufficient proportion of the total to justify the adoption of specific molecular screening strategies (Levy-Lahad *et al*, 1997; Peelen *et al*, 1997; Thorlacius *et al*, 1997; Moller *et al*, 2001). In the UK, there has been only limited evidence to date of founder mutations (Gayther *et al*, 1995,^1997^; Eccles *et al*, 1998; Lancaster *et al*, 1998; Peto *et al*, 1999), the most clear-cut example being BRCA1 *2800 delAA*, probably originating from the West of Scotland or Ireland (Liede *et al*, 2000). Since the present day populations of Scotland and Northern Ireland remain relatively homogeneous, and distinct from those of other parts of the UK (Bodmer *et al*, 1987; Liede *et al*, 2000), we have collated information on all BRCA1 and BRCA2 mutations recorded among families attending breast cancer genetics clinics in Aberdeen, Belfast, Dundee, Edinburgh or Glasgow. Additional data are included from a sequential series of male breast cancers presenting in South-East Scotland (Young *et al*, 2000). Findings are compared with those reported from England, Wales and elsewhere.

In both Scotland and Northern Ireland, families have tended to be large, at least until the current generation. They are also, typically, close-knit, maintaining contact even with members who have emigrated to distant parts of the world. Records of births, marriages and deaths are unusually complete and accessible (Collyer and DeMey, 1987) and the Scottish Cancer Registry, in particular, is recognised as among the best in the world (Kemp *et al*, 1985). For all these reasons, data on Scottish and Northern Irish families with genetic disorders can be extensive and accurate. The present data set, based on over 100 mutation-bearing families, is therefore of value for addressing questions about the clinical implications of BRCA1 and BRCA2 mutations.

## PATIENTS, FAMILIES AND METHODS

The study was approved by the Medical Research Ethics Committee for each of the regions in which clinical genetics services are based. The five centres mentioned above provide comprehensive National Health Service (NHS) clinical services for familial cancers, covering the entire population. They have collaborated closely in the design and development of these services for some 10 years and follow common policies on eligibility for regular surveillance by clinical/mammographic and ovarian screening (Haites *et al*, 2000). Molecular testing for BRCA1 or BRCA2 mutations was available only on a research basis from 1994 until 2001, when NHS funding was provided to establish a laboratory service accessible to all high-risk families. The majority of mutations included in this report were identified in one of the Scottish or Northern Irish research centres but some were found in family members tested at the Institute of Cancer Research, London (Dr Simon Gayther, Prof Michael Stratton), the University of Lund (Dr Ake Borg) or in centres in the USA, Australia, Iceland and the Netherlands. Mutation-detection methods therefore varied but, in general, they have been based on PCR amplification of gene fragments, covering most of the coding regions and splice sites, followed by SSCP or heteroduplex analysis on gels (Gayther *et al*, 1995,^1996^) or, more recently, by DHPLC (Wagner *et al*, 1999). Protein truncation assay (Hogervorst *et al*, 1995) has been applied on a limited scale and 150 samples from ‘high-risk’ families were screened specifically for the 6 kb duplication in BRCA1 *exon 13*, with one positive result (Mazoyer *et al*, 2000). Only some 20 ‘high-risk’ Scottish families have been included in a preliminary screen for large deletions in BRCA1. None has yet been found. Some centres have concentrated on screening those regions of both genes where mutations have already been identified in our population. This is likely to increase the apparent frequency of recurrent mutations but it is difficult to quantify the effect. The bulk of the screening was undertaken without this bias and, where priority was given to examination of particular PCR fragments, these, in total, represent a significant proportion of the entire coding regions of BRCA1 and BRCA2, so that nonrecurring mutations could be (indeed were) detected in the process. All mutations have been confirmed by direct sequencing and reconfirmed on a second blood sample from the index case before offering counselling and access to genetic testing for at-risk adult members of the family.

Family trees have been constructed, initially from information provided by families themselves. In most instances, they have filled out detailed family history forms, issued on referral to a genetics clinic. This normally entails a collaborative effort on the part of several family members, across the generations. Verification and extension of pedigrees can be undertaken by consulting public registration records (Collyer and DeMey, 1987) which, in comprehensive form, date from 1855. This facility, supported by a professional medical genealogist, is used selectively but when a germline mutation is detected, record tracing becomes a priority, since the process can establish links between two or more families already known to the clinics.

All practicable efforts are made to verify reported cancer diagnoses in the family, particularly if it seems probable that the pattern of disease is attributable to a high penetrance mutation. Confirmation can be obtained, in the case of deceased subjects, by consulting the Scottish or Northern Irish cancer registries (good data are available from mid-1960s onwards). For living patients, permission is sought to check relevant details from hospital records. In some instances, particularly for cancers that occurred over 40 years ago, supporting evidence can be obtained only from death certificates and, on occasions, there is no means of verification. A judgement then has to be made, on the basis of the family's own knowledge, whether or not to record a given relative as being affected by breast, ovarian or other cancers. When a mutation has been identified in one member of a given family, blood samples or tissue blocks are sought from affected relatives, to establish their mutation status. The process of tracing the distribution of the mutant allele within each family is still ongoing. In a number of cases, affected relatives have been shown not to carry the ‘family mutation’. These individuals are usually then reclassified as having sporadic cancer (with correspondingly reduced risk estimates for their descendants) but in two families a second mutation has been found and, in a few more, the pattern of cancers on both sides of the pedigree would be consistent with the presence of a second, still unidentified, mutation.

In the main, families selected for molecular testing met the published criteria (Haites *et al*, 2000) for, at least, ‘moderate risk’ (i.e. two close relatives with early onset breast or ovarian cancer) and most met the more stringent criteria defining ‘high risk’. Two exceptions were a single positive result from a consecutive series of women diagnosed with breast cancer under age 50 and one patient who had only a single relative with breast cancer, but an unusual familial cluster of early onset prostate, stomach and laryngeal cancers. She herself might have been discharged but was found to have breast cancer on her first clinic visit and a BRCA2 mutation was subsequently demonstrated. Twelve BRCA2 mutations have been identified among DNA samples from a consecutive series of 64 male breast cancers treated in Edinburgh since 1975. These samples were analysed, in the first instance, without reference to family history (Young *et al*, 2000). Most of the patients are deceased and construction of complete family trees is still proceeding, with the cooperation of their living relatives. However, two of them belong to families already registered with the regional cancer genetics clinic (because a cluster of cases among relatives had been recognised). In addition to the 12 from the consecutive series, a further 10 instances of male breast cancer have been recorded among the BRCA2 mutation-positive families in the present report. Two of these families included more than one affected male (three and two cases each).

## RESULTS

One hundred and nine mutations (61 in BRCA1, 48 in BRCA2) have been identified in 107 families. The vast majority cause truncation of the protein product (89 frame-shifts, six nonsense substitutions, eight splice site disruptions). One is a 6 kb duplication of BRCA1 *exon 13* and there are two instances of an intronic deletion believed to affect splicing (BRCA2 *9877-67 del4*). Only three missense substitutions are included. Two of these are recognised as pathogenic by the Breast Cancer Information Core (BIC) database and the third, BRCA1 *5242C*>*A*, although still listed as an unclassified variant, has been recorded 13 times in multicase breast cancer families.

Several mutations have been identified in more than one family each. Two are particularly frequent, BRCA1 *2800 delAA* and BRCA2 *6503 delTT*. The former has already been identified as a Scottish/Irish founder mutation (Liede *et al*, 2000), while the latter is also a recognised founder mutation, distributed throughout Europe and the USA (Mazoyer *et al*, 1996; Szabo and King, 1997; BIC database). In contrast to BRCA1 *2800 delAA*, the BRCA2 *6503 delTT* mutation is relatively frequent among families attending the Aberdeen and Dundee clinics (representing the North and East of Scotland), which may be in keeping with Scandinavian origin (Table 1

**Table 1:** Geographical distribution of BRCA1 and BRCA2 founder mutations

	BRCA1 *2800 delAA*	BRCA2 *6503 delTT*
Aberdeen	0	4
Dundee	1	4
Edinburgh	4	1
Glasgow	7	5
Belfast	2	0

Numbers of families with founder mutations BRCA1 *2800 delAA* or BRCA2 *6503 delTT* attending each of the five clinics in Scotland/Northern Ireland. Some families, with branches in different parts of the country, are recorded as attending more than one clinic. Clinics are listed in geographical sequence from North-East (Aberdeen) to South-West (Belfast).

).

**Figure 1:**
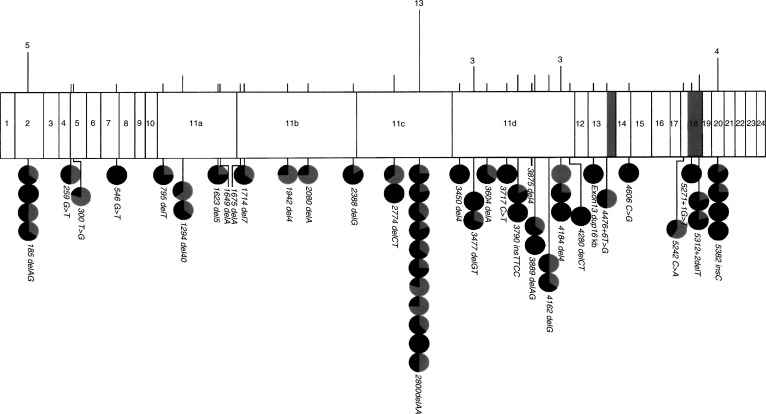
Diagrammatic representation of BRCA1 exons (plus intronic regions with pathogenic mutations, shown shaded). Vertical lines above the diagram represent all mutations detected, with heights proportional to numbers of cases. Actual numbers are given for most frequent mutations. Circles below the diagram represent families with at least three breast or ovarian cancers attributed to each mutation. The darker segment of each circle represents the proportion of breast cancers and the paler segment the proportion of ovarian cancers.

Eight further mutations, each detected at least three times, account for 27 families. Two of the three recognised Ashkenazi Jewish mutations (Levy-Lahad *et al*, 1997) –BRCA1 *185 delAG* and BRCA1 *5382 insC* –are among these eight; the third, BRCA2 *6174 delT*, was found on two occasions. The overall distribution of mutations is set out in Figure 1

.

In assessing the numbers of breast and ovarian cancers recorded in mutation-positive families, two families have been excluded (both with BRCA2 mutations). One was identified through the consecutive series of male breast cancers and no information has yet been obtained about relatives. The other has been counted as a BRCA1 mutation family, although a single BRCA2 mutation-carrier has been identified in one branch of the pedigree. That branch has not yet been investigated in detail. For the other family with two mutations (BRCA1 and BRCA2), it has been possible to identify the separate routes of inheritance and hence to calculate the numbers of breast and ovarian cancers attributable to each mutation. For all families, where an affected individual has been found not to carry the BRCA1 or BRCA2 mutation, that tumour has not been included in the calculations and, of course, only tumours occurring in blood relatives of mutation-positive family members are counted. Overall, comparable effort has been expended on gathering and verification of data on BRCA1 and BRCA2 mutation families. As shown in Table 2

**Table 2:** Breast and ovarian cancers recorded in BRCA1 and BRCA2 mutation-bearing families

	BRCA1 mutation +ve	BRCA2 mutation +ve
No. of families	61	46
No. of breast cancers	225	167
No. of ovarian cancers	94^a^	27^b^
Av. breast cancers/family	3.7	3.6
Av. ovarian cancers/fam	1.5	0.6
Ratio CaBr/CaOv	2.39:1	6.19:1

a Includes one primary peritoneal carcinoma.

b Includes one primary carcinoma of fallopian tube.

, the average number of breast cancers recorded per family is virtually the same for both mutations, but there are two and a half times as many ovarian cancers in BRCA1 families. The ratio of breast to ovarian cancers is 2.39 : 1 for BRCA1 families and 6.18 : 1 for BRCA2 families, a difference which is significant at the 0.01% level by *χ*^2^ analysis. This is consistent with data from the Breast Cancer Linkage Consortium (Ford *et al*, 1998) and other sources (Risch *et al*, 2001; Welcsh and King, 2001).

The mean age at diagnosis of breast cancer in BRCA1 mutation carriers was 42.6 years (95% CI 40.5–44.8). For BRCA2 mutation carriers (females only), it was 46.5 years (95% CI 43.6–49.4). Half of those with BRCA1 mutations were diagnosed at or below age 40, compared with just over one-quarter of those with BRCA2 mutations. For affected males (all with BRCA2 mutations), mean age at diagnosis of breast cancer was 59.4 years (95% CI 51.9–67.0). In keeping with the younger age at onset, 56% of BRCA1 families included at least one instance of bilateral disease, compared to 28% of BRCA2 families. (In the latter set, there was one male with bilateral breast cancers, onsets separated by 22 years.) Furthermore, in 36% of BRCA1 families, but fewer than 10% of BRCA2 families, there were two or more examples of bilaterality. Individual women with both breast and ovarian cancer were recorded in 36% of BRCA1 families, with up to three instances per family, whereas they occurred in only 10% of BRCA2 families and no family included more than one case. Ages of onset for ovarian cancer did not differ between families with BRCA1 or BRCA2 mutations (49.7 years, 95% CI 46.9–52.4 and 49.8 years, 95% CI 43.0–56.6 years, respectively). Over 90% of ovarian cancers, in both sets of families, were diagnosed at age 40 or older and half of the remainder were diagnosed at age 39.

A rather more contentious issue is the effect of mutation position, within either gene, on the relative risk of breast or ovarian cancer (Gayther *et al*, 1995; Iau *et al*, 2001; Thompson *et al*, 2001). For BRCA1, there is evidence that mutations towards the 3′ end are less likely to be associated with ovarian cancer (Gayther *et al*, 1995). We have subdivided our BRCA1 families as shown in Tables 3A, B

**Table 3:** Distribution of breast and ovarian cancers in families, according to site of (A) BRCA1 and (B) BRCA2 mutation

	(A) BRCA1 mutations 5′ of exon 12	BRCA1 mutations exons 12–24
Number of families	49	12
No. of breast cancers	161	64
No. of ovarian cancers	82	12
Average CaBr/family	3.3^a^	5.3^a^
Average CaOv/family	1.7	1.0
Ratio CaBr/CaOv	1.96:1^b^	5.33:1^b^
		
	**(B) BRCA2 mutations Within ‘OCCR’**	**BRCA2 mutations outside ‘OCCR’**
Number of families	25	21
No. of breast cancers	101	66
No. of ovarian cancers	21	6
Average CaBr/family	4.0	3.1
Average CaOv/family	0.84^c^	0.29^c^
Ratio CaBr/CaOv	4.81:1^d^	11:1^d^

a Difference in risks of breast cancer *P*=0.001 (by *χ*^2^).

b Difference in relative risks of breast *vs* ovarian cancer, *P*=0.0002 (by *χ*^2^).

c Difference in risks of ovarian cancer *P*<0.02 (by *χ*^2^).

d Difference in relative risks of breast *vs* ovarian cancer *P*<0.005 (by *χ*^2^).

, and confirm that the ratio of breast to ovarian cancers is strongly influenced by mutation site. Interestingly, our data suggest that this may result from both an increase in breast cancer risk and a reduction in ovarian cancer risk when the mutation occurs 3′ of exon 11.

**Figure 2:**
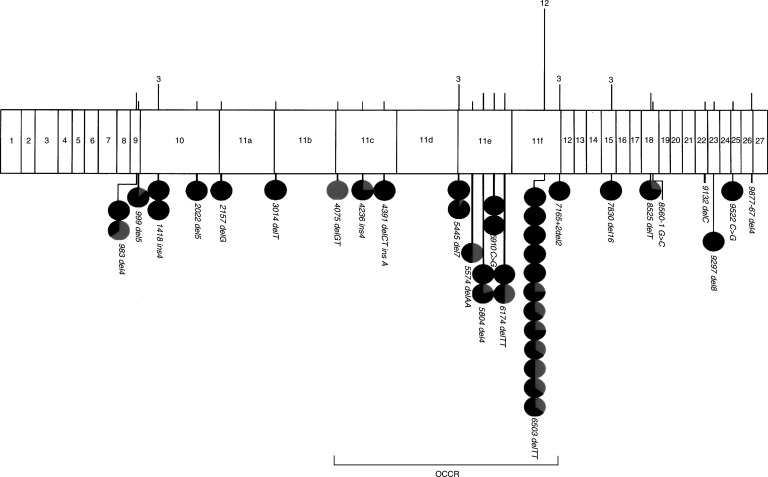
Diagram of BRCA2 exons, with the position of the ‘OCCR’ indicated. Annotation and symbols are as for Figure 1.

For BRCA2, it has been proposed that mutations within the central region of the gene, termed the ovarian cancer cluster region (‘OCCR’), are particularly associated with ovarian cancers (Figure 2

). Our data show that the ratio of breast to ovarian cancers is 4.81 : 1 for mutations within the OCCR and 11.0 : 1 for mutations 3′ and 5′ of that region. When the families identified only through the male breast cancer study are excluded, the trend for ovarian cancer remains in the same direction (4.04 breast cancers and 0.84 ovarian cancer per family within OCCR, 4.33 breast cancers and 0.50 ovarian cancers per family outside it; ratios 4.81 : 1 and 8.67 : 1, respectively) but the differences are no longer statistically significant.

## DISCUSSION

The proportion of BRCA2 mutation-bearing families in the present series (44%) is higher than that has been estimated indirectly from earlier (mainly much smaller) studies from the UK (Szabo and King, 1997; Lancaster *et al*, 1998). However, it is consistent with the prediction of roughly equal prevalence of BRCA1 and BRCA2 mutations, based on a survey of very early onset breast cancers (Peto *et al*, 1999). The authors of that survey noted that several UK studies have concentrated on multicase families in which ovarian cancer was prominent, thus overestimating the relative frequency of BRCA1 mutations. The International Breast Cancer Linkage Consortium data show that, among families with multiple cases of breast cancer but no known ovarian cancers, BRCA1 and BRCA2 mutations are almost equally represented (Ford *et al*, 1998). In the present series, there was no particular emphasis on ovarian cancer in selecting families for mutation testing but, of course, the clustering of ovarian with breast cancer in a family adds considerably to the prior probability of a mutation being present, as also does the occurrence of very early onset breast cancer. Account is taken of both these facts in the Scottish NHS guidelines for management of familial cancers (Haites *et al*, 2000), hence favouring ascertainment of BRCA1 mutation-bearing families. In most centres, facilities for BRCA1 mutation testing became available at least a year before those for BRCA2 so that, in consecutive series, there is some bias in favour of detecting BRCA1 mutations. This applies to the present data set, although the effect is probably not great and should diminish with time. On the other hand, our inclusion of 10 BRCA2 mutations detected only through a survey of male breast cancers obviously introduces a bias in the opposite direction. Nevertheless, it appears that in Scotland and Northern Ireland, the relative prevalence of BRCA2 mutations may be higher than in some other regions of Europe such as Norway (Moller *et al*, 2001), Sweden (Hakansson *et al*, 1997), Belgium (Claes *et al*, 1999) or France (Serova-Sinilnikova *et al*, 1997; Stoppa-Lyonnet *et al*, 1997).

Our findings do not permit any calculation of the absolute prevalence of BRCA1 or BRCA2 mutations in the Scottish and Northern Irish populations but they add to our knowledge of the distribution of specific mutations. Allowing for some bias in ascertainment, as discussed above, almost one-quarter of the families are accounted for by just two (BRCA1 *2800 delAA* and BRCA2 *6503 delTT*) and almost half by the 10 mutations that were found in at least three families each. Two of these are the well-recognised ‘Ashkenazi Jewish’ BRCA1 mutations (*185 delAG* and *5382 insC*), although at least one of the affected families is unaware of any Ashkenazi Jewish ancestry. The other recurring mutations are BRCA1 *3477 delGT* and *4184 del4*, BRCA2 *1418 ins4*, *5445 del7*, *7165*+*2 delT*, *7830 del16*. Most of these have been recorded many times in the BIC database and/or the Human Gene Mutation Database. Our earlier speculation (Liede *et al*, 2000) that BRCA1 *2800 delAA* may have originated in West-Central Scotland or in Ireland seems to be supported by the finding of three further cases, one from Glasgow and two from Belfast. BRCA2 *6503 delTT* has been found elsewhere in the UK as well as in Dutch, Swedish, Danish and Belgian families (BIC database). It was the most frequently recurring BRCA2 mutation (four examples) among 25 recorded by Gayther *et al* (1997) from ‘the United Kingdom and Eire’ but no further breakdown by geographical region was provided. BRCA1 *4184 del4* was identified as a recurring mutation in that survey and also in separate reports of families from Wales and the South of England (Lancaster *et al*, 1998; Eccles *et al*, 1998). In the last-mentioned study, BRCA1 *3875* was found five times in a total series of 17 mutations. It is entirely possible that further examples may be found of founder mutations associated with specific populations concentrated in particular regions of the UK but larger studies will be required. Scotland and Northern Ireland offer special advantages for this kind of analysis because of the relative population stability, the existence of an NHS-funded cancer genetics network and the excellence of genealogical and health-related records.

The proportion of all breast or breast/ovarian cancer families in our population attributable to recurring BRCA1 or BRCA2 mutations appears to be lower than in some other countries such as Iceland (Thorlacius *et al*, 1997), Israel (Levy-Lahad *et al*, 1997) or Norway (Moller *et al*, 2001), comparable to that in the Netherlands (Peelen *et al*, 1997; Claes *et al*, 1999), but much higher than in others, such as Italy (Szabo and King, 1997; Cippolini *et al*, 1999), and sufficient to have practical implications for the formal organisation of molecular screening. A first-stage analysis, covering the 10 most common BRCA1 and BRCA2 mutations, using multiplex PCR/DHPLC is rapid and relatively inexpensive. It can be offered to substantial numbers of families, then, if a negative result is obtained, more stringent risk criteria can be applied for complete analysis of the genes. This policy is already identifying further mutation-positive families (not included in the present report) among those who would not currently be eligible for full-scale molecular screening in our diagnostic laboratories (Haites *et al*, 2000). The occurrence of identical mutations in many families also provides opportunities for investigation of genetic and environmental factors that may modify penetrance or expression of the underlying mutation. This principle has been applied, for example, to identify an interaction between BRCA2 mutations and a polymorphism of RAD51 (Levy-Lahad *et al*, 2001; Wang *et al*, 2001). Among the families recorded here is one with seven cases of ovarian cancer over four generations, but no breast cancers. No other family with the same mutation, from the present series or from the BIC database, shows a comparable distribution of tumours and further research is warranted to establish whether this is merely a chance finding or a clue to an additional causal factor.

The influence of mutation site on relative risk of breast or ovarian cancer has been a controversial issue. For BRCA1, two studies published in 1995 indicated that the relative risk of ovarian cancer was substantially higher for mutations occurring in the 5′ two-thirds of the gene (Gayther *et al*, 1995; Shattuck-Eidens *et al*, 1995) but this was not confirmed in three other reports (Serova *et al*, 1996; Couch *et al*, 1997; Frank *et al*, 1998) and is not apparent in a fourth (Eccles *et al*, 1998), where the data are presented but not discussed in detail. Taking the 3′ end of exon 11 as the potential ‘change point’, we found a highly significant difference in the relative proportions of breast and ovarian cancers ‘upstream’ and ‘downstream’ of this position. Furthermore, the difference seems to arise both from an increased risk of breast cancer and a reduced risk of ovarian cancer for mutations in the 3′ third of the gene, although the difference in numbers of breast cancers per family does not achieve statistical significance on its own (*P*<0.1).

For BRCA2, an ‘Ovarian Cancer Cluster Region’ in the middle third of the gene was proposed by Gayther *et al* (1997). An international collaborative study (Neuhausen *et al*, 1998) provided some supporting evidence which, however, failed to reach statistical significance. In a more recent analysis of 164 BRCA2 mutation-bearing families (Thompson *et al*, 2001), 23.8% of tumours were ovarian (and 76.2% breast) among families with OCCR mutations: for families with mutations 5′ and 3′ of the OCCR, 8.8 and 9.6%, respectively, were ovarian cancers. Our own findings are similar but, whereas Thompson and colleagues found that a reduced absolute risk of breast cancer for OCCR mutations contributes substantially to the effect, in our series, the number of breast cancers per family is actually higher for OCCR *vs* non-OCCR mutations, and the entire increase in relative risk of ovarian cancer is attributable to a 2.9-fold higher rate of ovarian cancers in the former group. It will be important to determine which of these interpretations is correct in order to generate testable biological hypotheses to explain the findings.

The validity of our analysis depends on completeness of ascertainment of families and accuracy of breast and ovarian cancer diagnoses. There are obviously limitations on both counts but the large average family size, the accessibility of confirmatory records and the uniformity of our approach to extension and verification of family data allow some confidence in our data. Excluding nine families ascertained only through a series of male breast cancers (one such family was already excluded for lack of information) greatly reduces the statistical power of our analysis. It happens that all of these nine families carried mutations outside the OCCR and that none included any case of ovarian cancer. However, there is no indication that these are particular characteristics of BRCA2 mutations associated with male breast cancer. Six of the families with mutations inside the OCCR included affected males (two with more than one male case each) and two of these also included ovarian cancers.

Whether the associations between mutation position and phenotype are sufficiently strong to influence genetic counselling and management of affected families remains a moot point. Individual families, where the distribution of cancers seems to depart from the general rules, are not rare and, for the present, it may be wise to take at least as much account of the previous history of the family in question as of the statistical findings from large-scale surveys.

## Appendix 1.

E Anderson, J Campbell, R Cetnarskyj, S Drummond, I Kunkler, R Kurian, J Mackay, M Mackenzie, D Porter, M Porteous, E Smyth, M Steel, R Thomas, J Warner, I Young and D Young

Department of Clinical Oncology, Edinburgh Breast Unit, S.E. Scotland Regional Genetics Service, University of Edinburgh Medical School, Edinburgh, UK

V Anderson, C Beers, B CohenJ Scarborough and M Steel

School of Biology, University of St Andrews, Bute Medical Buildings, St Andrews KY16 9TS, UK

D Baty, D Goudie, L McLeish, M Reis, M Steel, A Thompson, D Young and J Yule

University of Dundee Medical School/Tayside Regional Genetics Service, Dundee, UK

C Bell, I Brown, B Gibbons, H Gregory, N Haites, K Kelly, B Milner, A Schofield and E Smyth

University of Aberdeen Medical School/Grampian Regional Genetics Service, Aberdeen, UK

D Black, M Boyd and A Renwick

Beatson Instiute for Cancer Research, University of Glasgow, UK

R Black, D Brewster and A Fordyce

Cancer Intelligence Unit and Scottish Cancer Registry, NHS Common Services Agency, Trinity Park house, Edinburgh, UK

A Bradley, C Graham, W Johnston, E Mallon and P Morrison

Department of Medical Genetics, Belfast City Hospital NHS Trust, Belfast, UK

S Cooke, R Davidson, F Douglas and M Whiteford

Duncan Guthrie Institute of Medical Genetics, University of Glasgow Medical School, Glasgow, UK

P Morrison

School of Biomedical Science, University of Ulster, Coleraine, UK

## References

[bib1] Bodmer JG, Kennedy LJ, Lindsay J, Wasik AM (1987) Applications of serology and the ethnic distribution of three locus HLA haplotypes. Br Med Bull 43: 94–121 3315104 10.1093/oxfordjournals.bmb.a072179

[bib2] Cippolini G, Ghimenti C, Sensi E, Iandolo A, Piccirilli A, Berti A, Naccarato G, Viacava P, Campani D, Bevilaqua G, Caligo MA (1999) Mutation analysis of BRCA1 and BRCA2 in Italian hereditary and sporadic forms of breast and ovarian cancers: tumor genotype–phenotype correlation in breast cancer BRCA-mutation carriers. Dis Markers 15: 101–102

[bib3] Claes K, Machackova E, DeVos M, Poppe B, DePaepe A, Messiaen L (1999) Mutation analysis of the BRCA1 and BRCA2 genes in the Belgian patient population and identification of a Belgian founder mutation. Dis markers 15: 69–73 10595255 10.1155/1999/241046 PMC3851655

[bib4] Collyer S, DeMay R (1987) Public records and recognition of genetic disease in Scotland. Clin Genet 31: 125–131 3568437 10.1111/j.1399-0004.1987.tb02782.x

[bib5] Couch FJ, DeShano ML, Blackwood MA (1997) BRCA1 mutations in women attending clinics that evaluate the risk of breast cancer. N Engl J Med 336: 1409–1415 9145677 10.1056/NEJM199705153362002

[bib6] Eccles DM, Englefield P, Soulby MA, Campbell IG (1998) BRCA1 mutations in Southern England. Br J Cancer 77: 2199–2203 9649133 10.1038/bjc.1998.366 PMC2150412

[bib7] Ford D, Easton DF, Stratton M, Narod SA, Goldgar D, Devilee P, Bishop DT, Weber B, Lenoir G, Chang-Claude J, Sobol H, Teare MD, Struewing J, Arason A, Scherneck S, Peto J, Rebbeck TR, Tonin P, Neuhausen S, Barkardottir R, Eyfjord J, Lynch H, Ponder BA, Gayther SA, Zelada-Hedman M (1998) Genetic heterogeneity and penetrance analysis of the BRCA1 and BRCA2 genes in breast cancer families. Am J Hum Genet 62: 676–689 9497246 10.1086/301749 PMC1376944

[bib8] Frank TS, Manley SA, Olopade OI, Cummings S, Garber JE, Bernhardt B, Antman K, Russo D, Wood ME, Mullineau L, Isaacs C, Peshkin B, Buys S, Venne V, Rowley PT, Loader S, Offit K, Robson M, Hampel H, Brener D, Winer EP, Clark S, Weber B, Strong LC, Thomas A (1998) Sequence analysis of BRCA1 and BRCA2: correlation of mutations with family history and ovarian cancer risk. J Clin Oncol 16: 2417–2425 9667259 10.1200/JCO.1998.16.7.2417

[bib9] Gayther SA, Harrington P, Russell P, Kharkevich G, Garkavtseva RF, Ponder BAJ, UKCCCR Familial Ovarian Cancer Study Group (1996) Rapid detection of regionally clustered germ-line BRCA1 mutations by multiplex heteroduplex analysis. Am J Hum Genet 58: 451–456 8644703 PMC1914578

[bib10] Gayther SA, Mangion J, Russell P Seal S, Barfoot R, Ponder BAJ, Stratton MR, Easton D (1997) Variation of risks of breast and ovarian cancer associated with different germline mutations of the BRCA2 gene. Nat Genet 15: 103–105 8988179 10.1038/ng0197-103

[bib11] Gayther SA, Warren W, Mazoyer S, Russell PA, Harrington PA, Chiano M, Seal S, Hamoudi R, van Rensburg EJ, Dunning AM, Love R, Evans G, Easton D, Clayton D, Stratton MR, Ponder BAJ (1995) Germline mutations of the BRCA1 gene in breast and ovarian cancer provide evidence for a genotype–phenotype correlation. Nat Genet 11: 428–433 7493024 10.1038/ng1295-428

[bib12] Haites N, Cancer Genetics Subgroup of the Scottish Cancer Group (2000) Guidelines for regional genetics centres on the implementation of genetic services for breast, ovarian and colorectal cancer families in scotland. CME J Gynaecol Oncol 5: 291–307

[bib13] Hakansson S, Johannsson O, Johannsson U, Sellberg G, Loman N, Gerdes AM, Holmberg E, Dahl N, Pandis N, Kristoffersson U, Olsson H, Borg A (1997) Moderate frequency of BRCA1 and BRCA2 germ-line mutations in Scandinavian familial breast cancer. Am J Hum Genet 60: 1068–1078 9150154 PMC1712420

[bib14] Hogervorst FBL, Cornelis RS, Bout M, van Vliet M, Oosterwijk JC, Olmer R, Bakker B et al (1995) Rapid detection of BRCA1 mutations by the protein truncation test. Nat Genet 10: 208–212 7663517 10.1038/ng0695-208

[bib15] Iau PTC, Macmillan RD, Blamey RW (2001) Germ line mutations associated with breast cancer susceptibility. Eur J Cancer 37: 300–321 11239752 10.1016/s0959-8049(00)00378-6

[bib16] Kemp I, Boyle P, Smans M, Muir CS (1985) An Atlas of Cancer in Scotland 1975–1980, Lyon, France: IARC publications No. 72

[bib17] Lancaster JM, Carney ME, Gray J, Myring J, Gumbs C, Sampson J, Wheeler D, France E, Wiseman R, Harper P, Futreal PA (1998) BRCA1 and BRCA2 in breast cancer families from Wales: moderate mutation frequency and two recurrent mutations in BRCA1. Br J Cancer 78: 1417–1420 9836472 10.1038/bjc.1998.701 PMC2063207

[bib18] Levy-Lahad E, Catane R, Eisenberg S, Kaufman B, Hornreich G, Lishinsky E, Shohat M, Weber BL, Beller U, Lahad A, Halle D (1997) Recurrent BRCA1 and BRCA2 mutations in Ashkenazi Jews in Israel: frequency and differential penetrance in ovarian cancer and in breast-ovarian cancer families. Am J Hum Genet 60: 1059–1067 9150153 PMC1712434

[bib19] Levy-Lahad E, Lahad A, Eisenberg S, Dagan E, Paperna T, Kasinetz L, Catane R, Kaufman B, Beller U, Renbaum P, Gershoni-Baruch R (2001) A single nucleotide polymorphism in the RAD51 gene modifies carrier risk in BRCA2 but not BRCA1 carriers. Proc Natl Acad Sci USA 98: 3232–3236 11248061 10.1073/pnas.051624098 PMC30636

[bib20] Liede A, Cohen BB, Black DM, Davidson RH, Renwick A, Hoodfar E, Olopade OI, Micek M, Anderson V, DeMey R, Fordyce A, Warner E, Dann JL, King M-C, Weber B, Narod SA, Steel CM (2000) Evidence of a founder BRCA1 mutation in Scotland. Br J Cancer 82: 705–711 10682686 10.1054/bjoc.1999.0984 PMC2363321

[bib21] Mazoyer S, Dunning AM, Serova O, Dearden J, Pugrt N, Healey CS, Gayther SA, Mangion J, Stratton MR, Lynch HT, Goldgar DE, Ponder BAJ, Lenoir GM (1996) A polymorphic stop codon in BRCA2. Nat Genet 14: 253–254 8896551 10.1038/ng1196-253

[bib22] Mazoyer S, the BRCA1 Exon 13 Duplication Screening Group (2000) The exon 13 duplication in the BRCA1 gene is a founder mutation present in geographically diverse populations. Am J Hum Genet 67: 202–212 PMC1287079 10827109

[bib23] Moller P, Borg A, Heimdal K, Apold J, Vallon-Christersson J, Hovig E, Maehls L, The Norwegian Inherited Breast Cancer Group, The Norwegian Inherited Ovarian Cancer Group (2001) The BRCA1 syndrome and other inherited breast or breast–ovarian cancers in a Norwegian prospective series. Eur J Cancer 37: 1027–1032 11334729 10.1016/s0959-8049(01)00075-2

[bib24] Neuhausen SL, Godwin AK, Gershoni-Baruch R, Schubert E, Garber J, Stoppa-Lyonnet D, Olah E, Csokay B, Serova O, Lalloo F, Osorio A, Stratton M, Offit K, Boyd J, Caligo MA, Scott RJ, Schofield A, Teugels E, Schwab M, Cannon-Albright L, Bishop T, Easton D, Benitez J, King M-C, Goldgar D (1998) Haplotype and phenotype analysis of nine recurrent BRCA2 mutations in 111 families: results of an international study. Am J Hum Genet 62: 1381–1388 9585613 10.1086/301885 PMC1377164

[bib25] Peelen T, Van Vliet M, Petrij-Bosch A, Mieremet R, Szabo C, van den Ouweland AM, Hogervorst F, Brohet R, Ligtenberg MJ, Teugels E, van der Hout AH, Gille JJ, Pals G, Jedema I, Olmer R, van Leeuwen I, Newman B, Plandsoen M, van de Est M, Brink G, Hageman S, Arts PJ, Bakker MM, Devile P (1997) A high proportion of novel mutations in BRCA1 with strong founder effects among Dutch and Belgian hereditary breast and ovarian cancer families. Am J Hum Genet 60: 1041–1049 9150151 PMC1712432

[bib26] Peto J, Collins N, Barfoot R, Seal S, Warren W, Rahman N, Easton DF, Evans C, Deacon J, Stratton MR (1999) Prevalence of BRCA1 and BRCA2 gene mutations in patients with early onset breast cancer. J Natl Cancer Inst 91: 943–949 10359546 10.1093/jnci/91.11.943

[bib27] Risch HA, McLaughlin JR, Cole DE, Rosen B, Bradley L, Kwan E, Jack E, Vesprini DJ, Kuperstein G, Abrahamson JL, Fan I, Wong B, Narod SA (2001) Prevalence and penetrance of germline BRCA1 and BRCA2 mutations in a population series of 649 women with ovarian cancer. Am J Hum Genet 68: 700–710 11179017 10.1086/318787 PMC1274482

[bib28] Serova O, Montagna M, Torchard D, Narod SA, Tonin P, Sylla B, Lynch HT, Feunteun J, Lenoir GM, (1996) A high incidence of BRCA1 mutations in breast–ovarian cancer families. Am J Hum Genet 58: 42–51 8554067 PMC1914944

[bib29] Serova-Sinilnikova OM, Boutrand L, Stoppa-Lyonnet D, Bressac-de-Paillerets B, Dubois V, Lasset C, Janin N, Bignon YJ, Longy M, Maugard C, Lidereau R, Leroux D, Frebourg T, Mazoyer S, Lenoir GM (1997) BRCA2 mutations in hereditary breast and ovarian cancer in France. Am J Hum Genet 60: 1236–1239 9150172 PMC1712433

[bib30] Shattuck-Eidens D, McClure M, Simard J, Labrie F, Narod SA, Couch F, Hoskins K, Weber B, Castilla L, Erdos M, Brody L, Friedman L, Ostermeyer E, Szabo C, King M-C, Jhanwar S, Offit K, Norton L, Gilewski T, Lubin M, Osborne M, Black D, Boyd M, Steel M, Ingles S, Haile R, Lindblom A, Olsson H, Borg A, Bishop DT, Solomon E, Radice P, Spatti G, Gayther S, Ponder B, Warren W, Stratton M, Liu Q, Fujimura F, Lewis C, Skolnick MH, Goldgar DE (1995) Collaborative survey of 80 mutations in the BRCA1 breast and ovarian cancer susceptibility gene. JAMA 273: 535–541 7837387

[bib31] Stoppa-Lyonnet D, Lauren-Puig P, Essioux L, Pages S, Ithier G, Ligot L, Forquet A, Salmon RJ, Clough KB, Pouillart P, Institut Curie Breast Cancer Group, Bonaiti-Pellie C, Thomas G (1997) BRCA1 sequence variation in 160 individuals referred to a breast/ovarian family cancer clinic. Am J Hum Genet 60: 1021–1030 9150149 PMC1712430

[bib32] Szabo C, King M-C (1997) Population genetics of BRCA1 and BRCA2. Am J Hum Genet 60: 1013–1020 9150148 PMC1712447

[bib33] Thompson D, Easton D, Breast Cancer linkage Consortium (2001) Variation in cancer risks, by mutation position, in BRCA2 mutation carriers. Am J Hum Genet 68: 410–419 11170890 10.1086/318181 PMC1235274

[bib34] Thorlacius S, Sigurdsson S, Bjarnadottir H, Olafsdottir G, Jonasson JG, Tryggvadottir L, Tulinius H, Eyfjord JE (1997) Study of a single BRCA2 mutation with a high carrier frequency in a small population. Am J Hum Genet 60: 1079–1084 9150155 PMC1712443

[bib35] Wagner T, Stoppa-Lyonnet D, Fleischmann E, Muhr D, Pages S, Sandberg T, Caux V, Moeslinger R, Langbauer G, Borg A, Oefner P (1999) Denaturing high performance liquid chromatography detects reliably BRCA1 and BRCA2 mutations. Genomics 62: 369–376 10644434 10.1006/geno.1999.6026

[bib36] Wang WW, Spurdle AB, Kolachana P, Bove B, Modan B, Ebbers SM, Suthers G, Tucker MA, Kaufman DJ, Dooby MM, Tarone RE, Daly M, Levavi H, Pierce H, Chetrit A, Yechezkel GH, Chenevix-Trench G, Offit K, Godwin AK, Struewing JP (2001) A single nucleotide polymorphism in the 5′ untranslated region of RAD51 and risk of cancer among BRCA1/2 mutation carriers. Cancer Epidemiol Biomark Prev 10: 955–960 11535547

[bib37] Welcsh P, King M-C (2001) BRCA1, BRCA2 and the genetics of breast and ovarian cancer. Hum Mol Genet 10: 705–713 11257103 10.1093/hmg/10.7.705

[bib38] Young IE, Kurian KM, MacKenzie MAF, Annink C, Black JMD, Kunkler IH, Cohen BB, Steel CM (2000) BRCA2 mutations in a Scottish male breast cancer population. Br J Cancer 83 (Suppl. 1): 25

